# Comparative evaluation of machine learning models for predicting *Cimbex quadrimaculata* population density across multiple problem formulations

**DOI:** 10.1371/journal.pone.0346494

**Published:** 2026-04-03

**Authors:** Yunus Güral

**Affiliations:** Faculty of Science, Department of Statistics, Firat University, Elazığ, Türkiye; Swedish Meteorological and Hydrological Institute, SWEDEN

## Abstract

The high variability and nonlinear relationships between environmental variables (such as temperature, relative humidity, and altitude) in ecological datasets prevent classical statistical models from obtaining accurate predictions. This study aimed to compare and investigate the performance of AI-based machine learning methods in analyzing complex ecological data structures. An agricultural dataset containing meteorological and vegetation variables was used as the representative case study. This dataset is based on population observations of *Cimbex quadrimaculata* in Diyarbakır (Eğil) and Elazığ (Keban) provinces in Türkiye between 2020 and 2022. Three different modeling approaches (binary classification, multiclass classification, and regression) were applied to the same data. This three-approach design enabled a systematic comparison of model performance, generalizability, and explainability on the same dataset using different definitions of the target variable. For classification tasks, the model performance was evaluated using accuracy, F1 score, and AUC metrics under a stratified 10-fold cross-validation scheme. Regression models, on the other hand, were assessed within a nested cross-validation framework using R², root mean square error (RMSE), mean absolute error (MAE). Ensemble-based boosting AI algorithms (Gradient Boosting, XGBoost, and LightGBM) demonstrated high accuracy and generalizability in characterizing the highly nonlinear relationships, nested effects, and non-additive interactions among multiple variables. Furthermore, the SHAP analysis improved the interpretability of the models and revealed that temperature- and humidity-related variables were consistently among the most influential predictors in the model predictions. Comparative performance evaluations of machine learning models showed that Gradient Boosting (94.3% accuracy, 0.983 AUC) and XGBoost (84.6% accuracy) were the strongest predictors in binary classification scenarios and overall analyses, respectively. In regression analyses, LightGBM and Random Forest algorithms stood out with cross-validation performances of approximately R² ≈ 0.73. In particular, the success of ensemble-based learning methods in capturing multidimensional relationships in ecological datasets explains the high predictive accuracy and robustness of these models across complex ecological data structures.

## 1. Introduction

Ecological and agricultural systems exhibit highly heterogeneous data structures due to the intricate interactions of environmental parameters and the resulting nonlinear dynamics. Multidimensional interactions between meteorological variables (temperature, relative humidity, altitude, wind speed, etc.) and biotic factors (plant age, canopy area, habitat characteristics, etc.) are fundamental determinants of the spatial and temporal distribution of pest populations. However, traditional parametric statistical methods are often insufficient in representing latent relationships between variables and producing highly accurate predictions when modeling such complex and stochastic data structures.

With recent advancements in data science, artificial intelligence and machine learning algorithms have emerged as powerful alternatives to conventional methods in environmental modeling processes. These modern approaches possess critical advantages, particularly in analyzing complex interactions in high-dimensional datasets, robustness to noisy data and high generalization capacity. Machine learning models enhance the operational effectiveness of agricultural decision support systems by enabling more precise simulation of ecological processes.

The literature shows that ensemble learning algorithms, particularly Random Forest, Gradient Boosting, XGBoost, and LightGBM, exhibit superior predictive performance in detecting nonlinear patterns in ecological datasets, as demonstrated by numerous empirical studies. In this context, *Cimbex quadrimaculata* Müller, 1766 (Hymenoptera: Cimbicidae), a pest species causing significant economic losses in almond cultivation in the Eastern and Southeastern Anatolia regions of Turkey [1], and exhibiting extreme sensitivity to climatic factors in its population dynamics, constitutes a critical focus for these advanced modeling approaches.

Field studies conducted in Diyarbakır and Elazığ provinces show that the annual fluctuations in the population density of this species are largely correlated with macro- and micro-climatic variables [2–6]. In this context, modeling the course of the pest population in an integrated manner with environmental factors is critically important for the development of sustainable integrated pest management strategies.

A review of the literature reveals that while various studies exist that utilize machine learning techniques in predicting pest populations, a large portion of these studies are limited to single modeling approaches or a limited number of algorithms. For example, Cebeci et al. [7] demonstrated strong relationships between *C. quadrimaculata* population density and temperature and humidity variables using Decision Tree and Random Forest algorithms. However, studies that conduct a simultaneous and systematic comparison of different machine learning paradigms (classification and regression) on the same ecological dataset are quite limited. This makes it difficult to fully understand the relative performance of algorithms on specific data structures. To fill this methodological gap, comparatively testing different modeling frameworks on the same empirical dataset will allow for a more holistic assessment of the ecological prediction capacity of AI-based approaches.

The main motivation of this study is to analyze the relationships between ecological variables affecting *Cimbex quadrimaculata* population density and machine learning algorithms using a systematic and comparative approach. Within the scope of this study, three different modeling scenarios were designed: binary classification, multiclass classification, and regression. This multifaceted methodology offers the opportunity to evaluate the predictive success, stability, and interpretability of algorithms within the same data ecosystem.

Accordingly, the main objectives of this study are:

- To analyze the relationships between ecological parameters and *Cimbex quadrimaculata* population density using different machine learning paradigms,
- To comparatively analyze the effects of different problem definitions such as binary classification, multiclass classification, and regression on model performance metrics,
- To determine the hierarchical performance of various artificial intelligence algorithms in terms of accuracy, generalizability, and model stability,
- To identify the specific contributions and importance levels of environmental and plant variables on model decisions using SHAP-based explainability analysis.

Overall, this study aims to reveal the strengths and weaknesses of different algorithms in the context of ecological data by addressing pest population density within a multidimensional modeling framework. The findings are expected to provide a generally valid comparative framework for modeling agricultural and environmental datasets of similar complexity.

## 2. Literature review

The use of machine learning methods in ecological data analysis has become a methodological standard in recent years. Since environmental parameters in ecological datasets often exhibit a nonlinear and multidimensional structure, classical statistical methods cannot adequately represent these complex relationships. Studies by Olden et al. [8], Thessen [9], and Schratz et al. [10] show that machine learning algorithms have become increasingly preferred in ecological modeling. Machine learning approaches offer significant advantages in ecological prediction studies due to their capacity to reveal complex interactions between variables in high-dimensional datasets.

The Random Forest algorithm, proposed by Breiman [11], is widely used in species distribution modeling studies due to its high accuracy rate and strong generalizability. Cutler et al. [12] showed that this algorithm exhibits superior prediction performance compared to traditional methods in ecological data analysis. Similarly, Prasad et al. [13] demonstrated that the Random Forest method provides high accuracy in classifying tree species. Elith et al. [14] and Peters et al. [15] state that ensemble-based methods such as boosted regression trees are effective tools for modeling complex interactions between environmental variables.

Boosting-based ensemble algorithms have been increasingly used in ecological data modeling studies in recent years. The XGBoost algorithm developed by Chen and Guestrin [16] is widely preferred due to its high computational efficiency and strong prediction performance on large datasets. The LightGBM algorithm developed by Ke et al. [17] is noteworthy for its fast learning capacity, especially on high-dimensional datasets. Studies by Pichler et al. [18], Miranda et al. [19], and Nicolas et al. [20] also show that these algorithms exhibit strong predictive performance on ecological datasets.

The use of machine learning methods in modeling the population dynamics of harmful species in agricultural ecosystems is increasing. Studies conducted by Pratheepa et al. [21] and Zhang et al. [22] demonstrate that the relationships between pest populations and temperature, relative humidity, and other meteorological variables can be successfully predicted using machine learning algorithms. In particular, decision tree and ensemble-based methods are reported to be effective in modeling nonlinear relationships between environmental variables and pest population density.

A review of the literature reveals that a large portion of the research is limited to evaluating a single modeling approach or a limited number of machine learning algorithms. Studies systematically comparing different algorithms on the same ecological dataset for multiple modeling problems (classification and regression) are quite limited. Therefore, comparative evaluation of different machine learning algorithms on the same dataset will contribute to a more comprehensive understanding of the performance of the approaches and the creation of stronger methodological frameworks for ecological data modeling studies.

## 3. Materials and methods

**Fig 1:**
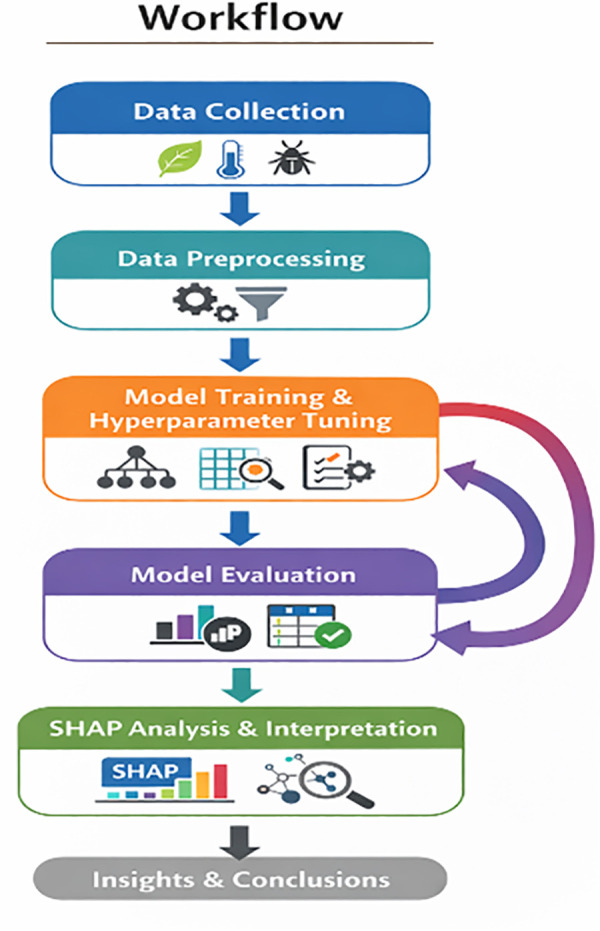
Workflow of the machine learning modeling process used in this study.

A comprehensive empirical dataset was created by recording *Cimbex quadrimaculata* larval density together with simultaneous meteorological and biotic parameters during field studies conducted in Elazığ and Diyarbakır between 2020 and 2022. These data served as the primary input for training, validating, and testing the machine learning models used in this study. The overall methodological workflow, including data preprocessing, model training, hyperparameter optimization, performance evaluation, and SHAP-based explainability analysis, is summarized in the flowchart presented in Fig. 1.

### 3.1. Dataset

The dataset used in this study included population observations of *Cimbex quadrimaculata* Müller, 1766 (Hymenoptera: Cimbicidae). Data were collected in field studies conducted between 2020 and 2022 at Elazığ (Keban, Nimri Village) and Diyarbakır (Eğil) as part of TÜBİTAK project number 3001-118O124. The main dataset, which contained 1,645 observations, was used for regression analyses. This dataset treats pest density as a continuous variable, and covers all locations and sampling years. Sub-datasets derived from the same main dataset were used for classification analyses. Within the scope of binary and multiclass classification analyses, the Eğil and Keban locations, as well as the wild and cultivated almond samples, were evaluated as separate subgroups. In this context, each sub-classification dataset contains approximately 410–415 observations.

The dependent variable was the number of pest larvae. The independent variables were temperature (°C), relative humidity (%), altitude (m), wind speed (m/s), tree age (years), crown projection (m^2^), and tree height (m). These represented a multidimensional ecological data structure that expresses the sensitivity of pest population to environmental and morphological factors. In this framework, the effect of meteorological and vegetation characteristics on pest density were evaluated, and the contribution of each variable was evaluated during the modeling process.

### 3.2. Data preprocessing and modeling approach

Missing values were imputed using the median because several variables exhibited non-symmetric distributions and the median provided a robust measure against outliers. Continuous variables were normalized to the range of [0,1] using min–max scaling to ensure comparability across features and support the joint use of distance-based, tree-based, and ensemble learning algorithms. All preprocessing steps were performed within the cross-validation framework to minimize the risk of data leakage.

Three approaches were used for modeling the dependent variable (pest population density):

1. Binary classification: A binary classification framework was applied by categorizing pest densities into low and high infestation levels.
2. Multiclass classification: Pest population density was divided into three hierarchical levels (low, medium, and high) to more precisely model infestation intensity. These categories represent relative infestation levels rather than precise biological thresholds.
3. Regression: Pest population density was treated as a continuous variable to capture both linear and nonlinear relationships with the ecological predictors.

This three-level approach enables a comprehensive and systematic comparison of classification and regression algorithms using the same dataset. Both parametric (Linear Regression, Ridge, Lasso, ElasticNet) and nonparametric methods (Decision Tree, Random Forest, Gradient Boosting, XGBoost, LightGBM, K-Nearest Neighbors (KNN), and Support Vector Regression (SVR)) were evaluated.

Regression models were trained and evaluated using a nested cross-validation framework. For the classification tasks, stratified 10-fold cross-validation was employed to preserve class distributions across folds. For models sensitive to hyperparameter selection, hyperparameter optimization was conducted using a randomized search strategy within an inner cross-validation loop (5-fold), while model performance was evaluated using an outer 10-fold cross-validation framework. For tree-based algorithms such as Random Forest, Gradient Boosting, XGBoost, and LightGBM, hyperparameters including the number of trees (n_estimators), maximum tree depth (max_depth), and learning rate were optimized. For the Support Vector Regression model, the kernel function and regularization parameters (C and gamma) were tuned, while for the K-Nearest Neighbors (KNN) algorithm the number of neighbors was optimized. The hyperparameter search was implemented within the cross-validation framework, allowing efficient exploration of the parameter space while reducing the risk of overfitting. This design ensures unbiased performance estimation and improves the generalizability of the models.

Model interpretability was evaluated using the SHAP (SHapley Additive exPlanations) method. SHAP is an explainability approach based on cooperative game theory that calculates the marginal contribution of each independent variable to the model prediction using Shapley values. This method provides a reliable and consistent explainability framework because it satisfies fundamental theoretical properties such as local accuracy, consistency, and missing feature status [23,24].

For tree-based models (Random Forest, Gradient Boosting, XGBoost, and LightGBM), the SHAP values were calculated using the TreeSHAP algorithm, which utilizes the internal structure of the model. This approach allows for the quantitative evaluation of the relative contribution of each variable to the prediction process while increasing the computational efficiency and preserving the theoretical Shapley properties.

### 3.3. Machine learning models

In this research, machine learning algorithms based on different mathematical principles were used to analyze the high-variance, multivariate, and nonlinear structures inherent in ecological data.

**Random Forest**

Random Forest is a powerful ensemble learning method based on the collective decision-making mechanism of numerous decision trees, integrating the principles of “bagging” (bootstrap aggregating) and random feature selection [11]. The algorithm combines the predictions of the trained trees by creating random subsets from the dataset, minimizing variance and increasing the generalizability of the model. The final prediction output ($\hat{y})$ is calculated by the arithmetic mean of the predictions of the individual trees,


$$\hat{y}=\frac{\text{1}}{T}\sum_{t=\text{1}}^{T}f_{t}(x)$$

Here, T represents the total number of trees in the model.

#### Gradient boosting.

Gradient boosting is an optimization strategy that minimizes the loss function by training weak learners (typically shallow decision trees) in a sequential manner [25]. This iterative approach, which defines the errors (residuals) of previous models as the target variable for the next model, gradually improves the predictive power of the model,


$$F_{m}(x)=F_{m-\text{1}}(x)+\gamma_{m}h_{m}(x)$$

Here, $h_{m}(x)$ represents the weak learner that focuses on errors in the current iteration, and $\gamma_{m}$ is the coefficient that controls the learning rate of the model.

#### Extreme gradient boosting.

XGBoost is an advanced algorithm that optimizes the classic gradient boosting method in terms of both computational efficiency and prediction accuracy [16]. The fundamental difference of the algorithm is that it adds a regularization term to the objective function to curb the tendency of complex models to overfit,


$$L=\sum_{i}l(y_{i},{\hat{y}}_{i})+\sum_{k}\Omega (f_{k})$$

Here, l represents the loss function, and $\sum_{k}\Omega (f_{k})$ represents the term that penalizes the complexity of the model, improving generalization.

#### LightGBM.

Offering computational efficiency on high-dimensional datasets, LightGBM uses histogram-based learning and a leaf-wise tree growth strategy [17]. Instead of level-based growth, continuing to grow from the leaf that minimizes loss allows the algorithm to capture complex patterns by building deeper trees,


$$L=\sum_{i}l(y_{i},{\hat{y}}_{i})$$

#### K-Nearest Neighbor.

KNN is a non-parametric algorithm based on the similarity principle of data points [26]. The prediction process is performed by averaging the values of k nearest neighbors in the data space:


$$\hat{y}=\frac{\text{1}}{k}\sum_{i\in N_{k}}y_{i}$$

This method offers a flexible modeling framework because it does not make any prior assumptions about the distribution of the dataset.

#### Linear and regularized regression models.

The linear regression model used as the base model in this study defines the relationship between the independent variables ($x_{j}$) and the dependent variable (y) through weighting coefficients (β):


$$y=\beta_{\text{0}}+\sum_{j=\text{1}}^{p}\beta_{j}x_{j}+\in$$

To manage the complexity of linear models and overcome multicollinearity problems, regularization methods such as Ridge and Lasso were preferred. These methods add penalty terms to the model to limit the regression coefficients:

- **Ridge Regression:** Reduces variance by penalizing the squares of the coefficients:

$$\lambda \sum_{j=\text{1}}^{p}\overset{\text{2}}{\underset{j}{\beta }}$$

- **Lasso Regression:** Performs variable selection by shrinking some coefficients to zero:

$$\lambda \sum_{j=\text{1}}^{p}|\beta_{j}|$$

### 3.4. Model evaluation metrics and explainability analysis

In order to objectively measure the predictive capacity and generalizability of the machine learning models developed within the scope of this research, standard metrics widely used in the literature for both classification and regression problems were employed. In addition, SHAP analysis was applied to enhance the interpretability of complex model structures and to quantitatively determine the marginal contribution of each variable to the predictions [24].

#### Classification performance metrics.

The performance of binary and multi-class classification models was evaluated based on accuracy, precision, recall (sensitivity), F1-score, and ROC-AUC [27,28].

**Accuracy**


$$\text{Accuracy}=\frac{TP+TN}{TP+TN+FP+FN}$$

**Precision**


$$\text{Precision}=\frac{TP}{TP+FP}$$

**Recall (Sensitivity)**


$$\text{Recall}=\frac{TP}{TP+FN}$$

**F1 Score**


$$F\text{1}=\text{2}\times \frac{\text{Precision}\times \text{Recall}}{\text{Precision}+\text{Recall}}$$

**ROC-AUC**


$$AUC=\int_{\text{0}}^{\text{1}}TPR(FPR)\;d(FPR)$$

In addition, Youden’s J index was used to determine optimal decision thresholds [29],


J=Sensitivity+Specificity−1

**Regression Performance Metrics**

In regression analyses where pest population density was treated as a continuous dependent variable, model performance was evaluated using the following metrics [30]:

**Coefficient of Determination (**$R^{\text{2}}$)


$$R^{\text{2}}=\text{1}-\frac{\sum (y_{i}-{\hat{y}}_{i}{)}^{\text{2}}}{\sum (y_{i}-\bar{y}{)}^{\text{2}}}$$

**Root Mean Square Error (RMSE)**


$$\text{RMSE}=\sqrt{\frac{\text{1}}{n}\sum_{i=\text{1}}^{n}(y_{i}-{\hat{y}}_{i}{)}^{\text{2}}}$$

**Mean Absolute Error (MAE)**


$$\text{MAE}=\frac{\text{1}}{n}\sum_{i=\text{1}}^{n}|y_{i}-{\hat{y}}_{i}|$$

These metrics provide complementary information regarding model accuracy, prediction error magnitude, and bias–variance trade-offs.

**Model Validation Strategy**

The classification models were evaluated using stratified 10-fold cross-validation, whereas the regression models were assessed using a nested cross-validation framework, providing a more robust estimation of model generalizability.

**Model Explainability: SHAP Analysis**

The SHAP method, based on cooperative game theory, was used to interpret the local and global behavior of machine learning models [24]:


$$\varphi_{i}=\sum_{S\subseteq N\backslash \{i\}}\frac{|S|!(|N|-|S|-\text{1})!}{|N|!}\left[f(S\cup \{i\})-f(S)\right]$$

Here, $\varphi_{i}$ represents the contribution of the relevant variable, S represents feature subsets, and f(S) denotes the model output.

The optimal hyperparameter values selected for each regression model using RandomizedSearchCV are presented in Table 1. These parameters represent the configurations that maximized model performance within the nested cross-validation framework. Hyperparameter tuning was performed within the inner loop of the nested cross-validation framework to avoid information leakage and ensure unbiased model evaluation.

**Table 1:** Optimal hyperparameter values for regression models.

Model	Optimal hyperparameters
Random Forest	n_estimators = 600; min_samples_split = 5; min_samples_leaf = 1; max_features = sqrt
LightGBM	num_leaves = 127; learning_rate = 0.05; n_estimators = 600; subsample = 1.0; colsample_bytree = 0.8
Decision Tree	max_depth = None; min_samples_split = 20; min_samples_leaf = 8
XGBoost	n_estimators = 600; max_depth = 5; learning_rate = 0.05; subsample = 0.7; colsample_bytree = 0.8
Gradient Boosting	n_estimators = 400; learning_rate = 0.05; max_depth = 3; subsample = 0.85
KNN	n_neighbors = 7; weights = distance; p = 2
SVR	C = 10; gamma = auto; epsilon = 0.2
Ridge Regression	alpha = 1.27
Linear Regression	No hyperparameter tuning applied
ElasticNet	alpha = 0.01; l1_ratio = 0.55
Lasso Regression	alpha = 0.078

All analyses were conducted using Python version 3.10. Model development employed scikit-learn library [31], XGBoost [16], and LightGBM [17]. Data preprocessing and visualization were performed using NumPy, Pandas, and Matplotlib.

## 4. Results

Three modeling approaches, binary classification, multiclass classification, and regression, were used in this study to explore the relationship between *Cimbex quadrimaculata* population density and a set of ecological variables and to compare the predictive performance of different machine learning algorithms. In general, ensemble-based algorithms yielded higher predictive scores compared to single-model approaches.

### 4.1. ROC analysis and binary classification results

The infestation risk thresholds determined using the ROC analysis and Youden’s J index were presented Table 2. In the wild almond samples from Eğil, higher predicted risk values were observed at temperatures below 30.2°C and relative humidity levels above 68%, based on model-derived classification scores. In contrast, samples from Keban exhibited a higher risk of infestation at relatively low temperatures and humidity values. These results indicate location-specific differences in the statistical decision thresholds associated with the infestation risk.

**Table 2:** Risk direction analysis based on cut-off values.

	Eğil Wild Almond	Keban Wild Almond	Eğil Cultivated Almond	Keban Cultivated Almond	All Trees
Feature	Risk Direction	Risk Direction	Risk Direction	Risk Direction	Risk Direction
Temperature	Risk < 30.20	Risk < 27.30	Risk < 31.60	Risk < 26.90	Risk < 29.4
Humidity	Risk > 68.30	Risk < 48.40	Risk > 62.20	Risk < 42.90	Risk > 48.2
Altitude	Risk < 852.00	Risk < 1390	–	–	Risk < 1381.0
Wind Speed	–	–	Risk < 2.00	–	–
Tree Age	Risk < 20.00	–	–	Risk < 6.00	–
Tree Height	Risk < 4.20	Risk < 3.90	–	Risk < 2.30	–
Tree Crown	Risk > 2.40	Risk < 2.50	–	–	–

It should be noted that these cutoff values represent statistical decision thresholds derived from ROC analysis and should not be interpreted as biological or ecological limits.

Table 3 presents the performance metrics of machine learning models trained with the binary classification approach. The findings show a distinct performance hierarchy among the prediction capabilities of the algorithms used. In particular, the Gradient Boosting algorithm exhibited the highest performance, achieving an accuracy of 94.3% and an AUC of 0.983, indicating a strong ability to distinguish between classes. Similarly, the Random Forest and XGBoost models also demonstrated high classification performance, with accuracy values ranging from 91% to 93%. In contrast, traditional models based on linear relationships showed comparatively lower performance in terms of both accuracy and AUC values.

**Table 3:** Performance metrics of binary classification models.

	Model	Accuracy	Precision	Recall	F1 Score	AUC
Eğil Wild Almond	Decision Tree	0.797	0.745	0.729	0.737	0.823
	Random Forest	0.902	0.860	0.896	0.878	0.943
	Gradient Boosting	0.943	0.902	0.958	0.929	0.983
	XGBoost	0.902	0.833	0.938	0.882	0.964
Keban Wild Almond	Decision Tree	0.837	0.826	0.877	0.851	0.875
	Random Forest	0.862	0.833	0.923	0.876	0.957
	Gradient Boosting	0.837	0.857	0.831	0.844	0.949
	XGBoost	**0.894**	**0.871**	**0.938**	**0.904**	**0.952**
Eğil Cultivated Almond	Decision Tree	0.634	0.736	0.557	0.634	0.675
	Random Forest	0.764	0.806	0.771	0.788	0.845
	Gradient Boosting	0.821	0.843	0.843	0.843	0.866
	XGBoost	0.789	0.797	0.843	0.819	0.860
Keban Cultivated Almond	Decision Tree	0.680	0.851	0.685	0.759	0.696
	Random Forest	0.704	0.802	0.793	0.798	0.736
	Gradient Boosting	**0.752**	**0.843**	**0.815**	**0.829**	**0.782**
	XGBoost	0.736	0.824	0.815	0.820	0.770
All Trees	Decision Tree	0.688	0.695	0.738	0.716	0.749
	Random Forest	0.818	0.819	0.844	0.831	0.900
	Gradient Boosting	0.812	0.822	0.825	0.824	0.887
	XGBoost	0.846	0.842	0.875	0.858	0.910

This difference among the models can be attributed to the methodological advantages of ensemble learning methods in capturing nonlinear and complex variable interactions inherent in ecological datasets. In particular, the iterative learning strategy of the Gradient Boosting algorithm enables the model to minimize errors sequentially and produce more reliable predictions in dynamic processes such as pest population density.

### 4.2. MultiClass classification performance and variable effects

Findings from multiclass classification analyses reveal that the XGBoost model exhibits superior performance, achieving the highest accuracy values across all subgroups (Table 4). The model’s classification accuracy was recorded as 85.4% in Eğil wild almond samples, 81% in the Keban location, and 71% in Eğil cultivated almonds, while the overall evaluation including all trees reached 78%. These results indicate that the algorithm demonstrates consistent and generalizable predictive capability under different ecological conditions.

**Table 4:** Classification report for the best multiclass model.

	Class	Precision	Recall	F1-Score
Eğil Wild Almond	Clean	0.85	0.96	0.90
	Medium	0.79	0.63	0.70
	High	0.89	0.89	0.89
	Accuracy	–	–	0.85
	Macro Avg	0.84	0.83	0.83
	Weighted Avg	0.85	0.85	0.85
Keban Wild Almond	Clean	0.90	0.97	0.93
	Medium	0.70	0.64	0.67
	High	0.70	0.64	0.67
	Accuracy	–	–	0.81
	Macro avg	0.77	0.75	0.76
	Weighted avg	0.80	0.81	0.81
Eğil Cultivated Almond	Clean	0.76	0.81	0.79
	Medium	0.60	0.58	0.59
	High	0.83	0.50	0.62
	Accuracy	–	–	0.71
	Macro Avg	0.73	0.63	0.67
	Weighted Avg	0.71	0.71	0.70
All Trees	Clean	0.84	0.86	0.85
	Medium	0.64	0.61	0.62
	High	0.82	0.83	0.83
	Accuracy	–	–	0.78
	Macro Avg	0.77	0.77	0.77
	Weighted Avg	0.78	0.78	0.78

When class-based F1-scores are examined, the model shows stronger discriminatory power, particularly in the “clean” and “high” categories. In the Eğil wild almond dataset, the F1-scores for these classes reached 0.90 and 0.89, respectively. In contrast, the F1-scores for the “medium” category remained in the 0.59–0.70 range, indicating the relative difficulty in modeling this class. This limitation may be attributed to the overlap of medium-level infestation with adjacent classes, as well as the ambiguity of boundaries within transitional ecological conditions.

Furthermore, the high recall values (up to 0.97) obtained for the “clean” class demonstrate the model’s effectiveness in identifying low-risk or pest-free conditions. On the other hand, the lower recall and F1-scores observed in the “medium” and “high” classes of cultivated almonds suggest that ecological variability and class imbalance may influence model performance. Overall, while the XGBoost algorithm exhibits strong performance in multiclass classification, the continuity of ecological processes and the absence of clear boundaries between infestation levels make accurate classification—particularly at intermediate levels—more challenging.

**Fig 2:**
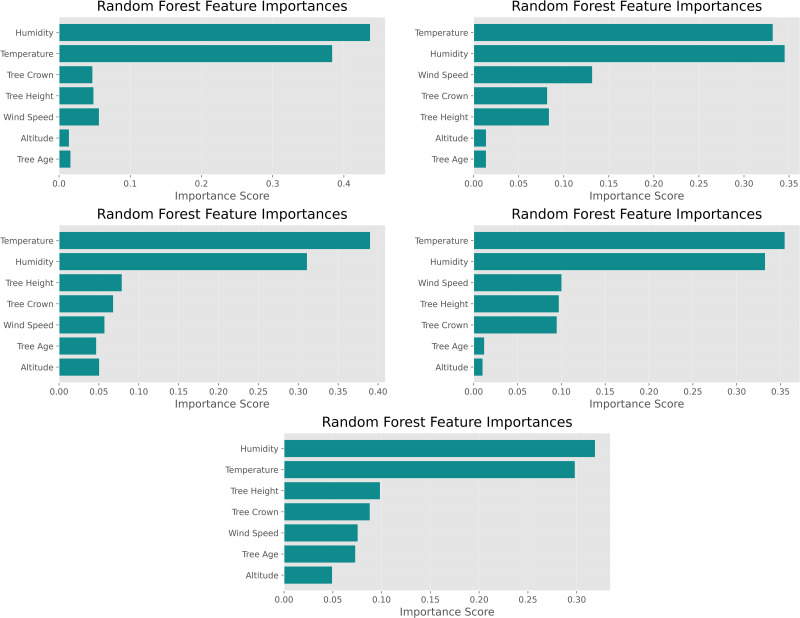
Feature importance scores from random forest classification.

When the variable importance levels obtained from the Random Forest algorithm are examined, relative humidity and temperature emerge as the most influential predictors of the model’s performance across all subgroups (Fig 2). These variables are followed by crown projection area and tree height, while altitude and tree age show comparatively lower contributions. SHAP analyses further confirm this ranking, indicating that the mean absolute SHAP values of relative humidity and temperature are substantially higher than those of the other variables.

These findings suggest that temperature and relative humidity play a dominant role in shaping model decision boundaries, particularly in multiclass classification scenarios. The prominence of these climatic variables can be attributed to their direct influence on insect physiology, life cycle, and reproductive dynamics, which are key drivers of pest population fluctuations. Overall, the results are consistent with ecological studies highlighting temperature and humidity as the primary determinants of pest population development.

**Fig 3:**
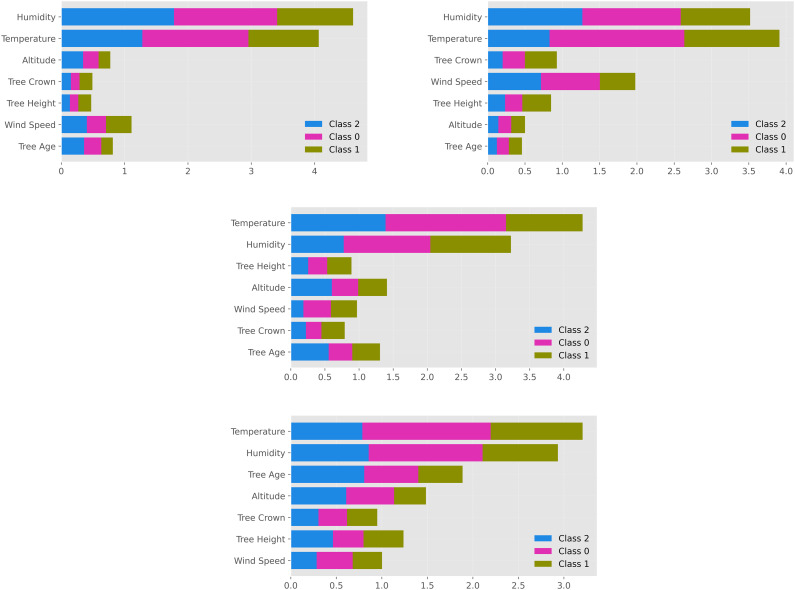
SHAP value summary plot for XGBoost multiclass classification of infestation levels.

The SHAP analysis results presented in Fig 3 demonstrate that relative humidity and temperature have the highest mean absolute SHAP values across all datasets and play a dominant role in model predictions. In wild almond data from the Eğil and Keban locations, these two variables consistently occupy the top two positions; crown projection area and altitude show moderate contributions, while wind speed and tree age exhibit relatively lower effects.

Location-based variations in the importance of secondary variables are also evident. For example, in the Keban dataset, wind speed and tree age contribute more prominently to model outputs, whereas in the Eğil dataset, altitude and crown projection area become more influential. A similar pattern is observed in the Eğil cultivated almonds and the combined dataset including all tree groups, where relative humidity and temperature consistently retain their dominant roles.

This consistency across different modeling scenarios indicates that the models learn stable patterns across data subsets and exhibit strong generalization capability. The persistent dominance of relative humidity and temperature suggests that the model’s decision-making process is reliably structured around these key variables and effectively captures the underlying relationships within the dataset.

### 4.3. Regression results and model-based variable ranges

In the regression analysis, pest population density was defined as a continuous dependent variable, and the generalizability of the models was evaluated using a nested cross-validation strategy (10-fold outer and 5-fold inner loops) incorporating hyperparameter optimization. The reported CV R² values correspond to the mean performance across the outer folds, ensuring an unbiased estimation of model generalizability. The performance metrics presented in Table 5 reflect the conservative and robust framework provided by this methodological approach for assessing model performance.

**Table 5:** Performance comparison of regression models.

Model	R²	RMSE	CV R² (mean ± std)	CV RMSE (mean ± std)	CV MAE (mean ± std)
Random Forest	0.840	2.72	0.721 ± 0.190	3.66 ± 2.68	1.73 ± 0.27
LightGBM	0.838	2.73	0.731 ± 0.172	3.65 ± 2.63	1.82 ± 0.25
Decision Tree	0.793	3.09	0.570 ± 0.226	4.52 ± 2.68	1.96 ± 0.31
Gradient Boosting	0.750	3.39	0.538 ± 0.498	4.36 ± 2.86	2.01 ± 0.18
XGBoost	0.740	3.47	0.557 ± 0.457	4.30 ± 2.85	1.91 ± 0.27
KNN	0.336	5.53	0.589 ± 0.151	4.43 ± 2.44	2.33 ± 0.25
SVR	0.265	5.82	0.487 ± 0.142	4.96 ± 2.47	2.61 ± 0.38
Ridge Regression	0.228	5.97	0.150 ± 0.078	6.28 ± 2.26	4.20 ± 0.33
Linear Regression	0.227	5.97	0.149 ± 0.079	6.28 ± 2.26	4.21 ± 0.33
ElasticNet	0.139	6.30	0.150 ± 0.078	6.28 ± 2.26	4.20 ± 0.33
Lasso Regression	0.120	6.37	0.149 ± 0.078	6.28 ± 2.26	4.20 ± 0.33

CV R² = Cross-Validation R²; RMSE = Root Mean Square Error; MAE = Mean Absolute Error.

According to the findings, the LightGBM algorithm exhibited the highest predictive performance with an average coefficient of determination (CV R² = 0.731 ± 0.172), followed closely by the Random Forest model (CV R² = 0.721 ± 0.190). The superior performance of these ensemble-based methods compared to linear and kernel-based approaches highlights their effectiveness in capturing nonlinear patterns and multidimensional interactions in ecological datasets. Although Random Forest produced lower error values in some folds (CV RMSE = 3.66), LightGBM demonstrated a more balanced profile in terms of generalizability and model stability.

The standard deviations observed in CV R² values indicate that model performance is influenced by the heterogeneous structure of the dataset and the stochastic variability inherent in ecological systems. This suggests that, despite the overall strong performance of the models, predictive relationships remain sensitive to data distribution. In contrast, single decision trees and k-nearest neighbor methods showed moderate performance (CV R² = 0.570–0.589), whereas linear regression, Ridge, Lasso, and ElasticNet models performed substantially worse (CV R² ≈ 0.15). These results clearly demonstrate the limitations of classical linear approaches in representing the complex and interactive variable structures inherent in ecological systems.

**Fig 4:**
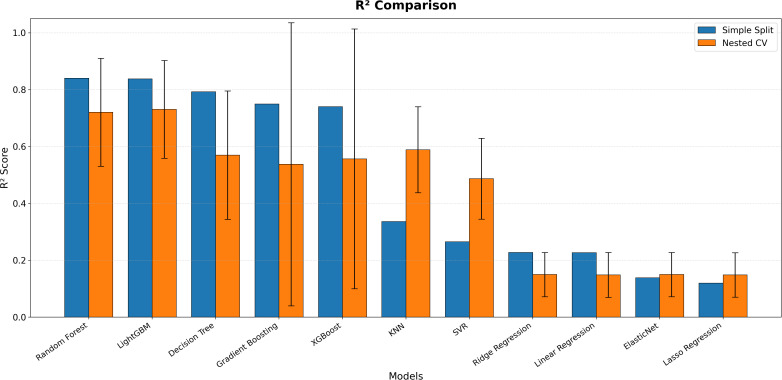
Performance comparison of regression models.

The comparative analyses presented in Fig 4 reveal the clear impact of the methodological approach used in model performance evaluation—simple splitting and nested cross-validation—on the results. For all models, the R² values obtained using the simple splitting method were systematically higher than those obtained with nested cross-validation. This suggests the presence of an optimistic bias in model evaluation when using simple data splitting. In contrast, the nested cross-validation approach provides a more cautious and realistic assessment of model generalizability by evaluating performance on previously unseen data and reducing the risk of overfitting. In particular, ensemble-based algorithms such as Random Forest, LightGBM, and XGBoost demonstrated robust performance by maintaining higher and more stable results across both evaluation strategies compared to other models.

**Fig 5:**
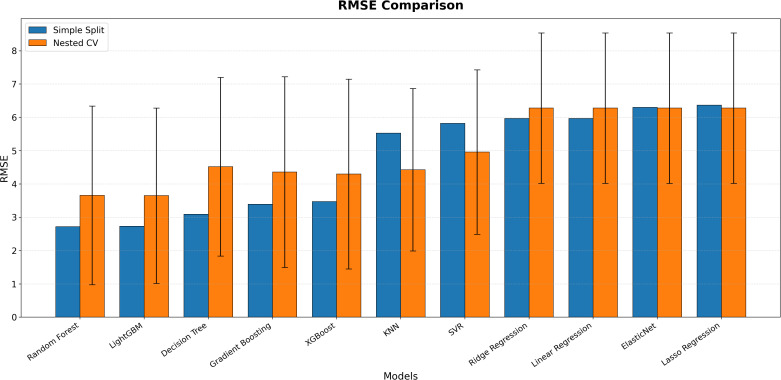
RMSE comparison of regression models.

Fig 5 shows that the RMSE values, representing model prediction errors, follow a pattern consistent with the R² findings. The generally higher error values under nested cross-validation indicate that this approach evaluates model performance under stricter conditions and presents prediction errors on a more realistic scale. The results clearly demonstrate that ensemble methods maintain their predictive superiority with lower RMSE values, whereas linear and single decision tree models exhibit higher error margins. Overall, the combined interpretation of both figures highlights that nested cross-validation represents a reliable methodological framework for obtaining robust and generalizable results in ecological data modeling.

Table 6 summarizes the variable ranges within which the regression model predicted relatively low pest population densities across the observed data space. The “Minimum Mean Predicted Pest Number” values represent the model estimates for observations corresponding to these parameter combinations; however, these results should not be interpreted as biological or ecological optimum conditions.

**Table 6:** Variable ranges associated with lower predicted pest population density.

Variable	Variable Range	Minimum Mean Predicted Pest Count	Number of Observations
Humidity	(20.60–21.30)	0.63	45
Temperature	(34.10–36.70)	1.48	90
Tree Age	(6.00–8.00)	2.66	275
Tree Crown Projection	(1.20–1.30)	2.69	55
Tree Height	(2.10–2.20)	3.08	137
Altitude	(1381.00–1385.00)	2.66	275
Wind Speed	(3.88–4.04)	4.96	50

A detailed examination of the model outputs shows that lower density predictions are concentrated within relatively narrow ranges of key microclimatic variables, particularly relative humidity (20.60–21.30%) and temperature (34.10–36.70 °C). Similarly, reduced pest density predictions were observed within specific intervals of dendrometric variables, including tree age (6–8 years), canopy projection area (1.20–1.30 m²), and tree height (2.10–2.20 m). This indicates that the model captures localized patterns in which pest populations tend to decrease under certain combinations of environmental and structural conditions.

In contrast, higher values of altitude and wind speed were associated with increased pest density predictions. However, these relationships should be interpreted as model-based statistical associations rather than causal effects. Furthermore, the relatively limited number of observations within some variable ranges (e.g., relative humidity and canopy projection area) may constrain the robustness and generalizability of these findings. Therefore, these results should be considered descriptive outputs of the model and should not be directly used for pest management or ecological decision-making without further biological validation.

Relative humidity and temperature variables were represented with higher model contribution values than other variables across all binary classification, multiclass classification and regression analyses. The SHAP summary plots and feature importance rankings showed that these two variables were ranked highest in all analysis scenarios (Figs 2 and 3). However, the contributions of other environmental and vegetative variables varied depending on the modeling approach and location. Overall, these findings further support the strong predictive capability of ensemble-based models in capturing key drivers of pest population dynamics in complex ecological datasets.

## 5. Discussion

In this study, the population density of *Cimbex quadrimaculata* was modeled using machine learning algorithms under three different problem definitions (binary classification, multi-class classification, and regression) on the same dataset. This holistic approach allowed for a systematic comparison of different modeling architectures in terms of predictive capacity, generalizability, and interpretability.

The empirical findings demonstrate that ensemble-based algorithms—especially Gradient Boosting and XGBoost—show a clear advantage over linear and single-tree models in classification problems. These results indicate that complex and nonlinear variable interactions inherent in ecological datasets can be captured more effectively by ensemble methods. Indeed, the literature frequently highlights the advantages of ensemble learning approaches in modeling complex relationships among environmental variables [12,14]. Furthermore, the high computational efficiency and strong predictive capability of the XGBoost algorithm have led to its widespread use in analyzing such complex ecological patterns. The relatively lower performance observed in the “medium” density class in multiclass classification analyses can be explained not by structural limitations of the model, but rather by statistical uncertainties and overlaps between classes in transition zones.

The strong cross-validation performance of Random Forest and LightGBM in regression analyses confirms the effectiveness of these methods in modeling complex relationships involving continuous variables. In particular, the nested cross-validation approach used in this study provides more conservative yet more reliable performance estimates compared to simple data splitting methods, which is critical for evaluating model generalizability. In this context, as also emphasized by Schratz et al. [10], the use of advanced validation strategies is essential given the high variability and heterogeneity of ecological datasets.

Within the framework of model explainability, SHAP and feature importance analyses revealed that temperature and relative humidity consistently had the highest contributions across all modeling approaches. This finding is consistent with ecological literature emphasizing the key role of climatic variables in shaping pest population dynamics. However, it is important to note that these contributions should not be interpreted as direct causal relationships, but rather as statistical patterns learned by the models. The variation in the influence of plant-related variables across locations further indicates that local environmental conditions modulate the relationships captured by the models.

Finally, the variable ranges associated with lower pest density represent descriptive patterns identified within the dataset. However, interpreting these values as biological optima or direct management thresholds may be misleading. The limited number of observations in some parameter ranges also constrains the generalizability of these findings. Therefore, additional validation studies using independent datasets and incorporating temporal dynamics are necessary to enhance the applicability of these models in decision support systems.

## 6. Conclusion

This study demonstrates that machine learning-based approaches provide robust exploratory and comparative tools for analyzing nonlinear and multivariate ecological data structures. Evaluating different problem definitions and algorithms on the same dataset reveals the sensitivity of model performance to the problem definition and validation strategy.

The results indicate that ensemble-based algorithms outperform traditional approaches across different modeling frameworks. In classification tasks, Gradient Boosting achieved the highest performance (accuracy = 94.3%, AUC = 0.983), while XGBoost showed the best overall performance across all datasets (accuracy = 84.6%). In regression analyses, LightGBM and Random Forest demonstrated higher generalization performance (CV R² ≈ 0.731 and 0.721, respectively), whereas linear and regularized models showed substantially lower performance (CV R² ≈ 0.15).

Across all modeling approaches, temperature and relative humidity consistently showed the highest contribution to model predictions, highlighting their dominant role in shaping pest population dynamics within the dataset. These findings demonstrate that ensemble learning methods provide a more robust and reliable framework for capturing complex ecological interactions compared to traditional statistical approaches.

This study contributes methodologically by integrating multiple problem formulations—binary classification, multiclass classification, and regression—within the same dataset, providing a comprehensive perspective on how algorithm selection and problem definition influence model performance in ecological modeling.

Future research incorporating independent validation datasets, temporal dynamics, and multi-scale analyses will further enhance the generalizability and practical applicability of these models.

## Supporting information

S1 Data Dataset used in this study. (XLSX)
